# The Association between Hematological Parameters and Insulin Resistance Is Modified by Body Mass Index – Results from the North-East Italy MoMa Population Study

**DOI:** 10.1371/journal.pone.0101590

**Published:** 2014-07-07

**Authors:** Rocco Barazzoni, Gianluca Gortan Cappellari, Annamaria Semolic, Enrico Chendi, Mario Ius, Roberta Situlin, Michela Zanetti, Pierandrea Vinci, Gianfranco Guarnieri

**Affiliations:** Internal Medicine, Department of Medical, Surgical and Health Sciences, University of Trieste, Trieste, Italy; Weill Cornell Medical College Qatar, Qatar

## Abstract

**Objective:**

Increments in red blood cell count (RBC), hemoglobin (Hb) and hematocrit (Ht) levels are reportedly associated with higher insulin resistance (IR). Obesity may cause IR, but underlying factors remain incompletely defined, and interactions between obesity, hematological parameters and IR are incompletely understood. We therefore determined whether: 1) BMI and obesity per se are independently associated with higher RBC, hemoglobin and hematocrit; 2) hematological parameters independently predict insulin resistance in obese individuals.

**Design and Methods:**

We investigated the associations between BMI, hematological parameters and insulin resistance as reflected by homeostasis model assessment (HOMA) in a general population cohort from the North-East Italy MoMa epidemiological study (M/F = 865/971, age = 49±1).

**Results:**

In all subjects, age-, sex- and smoking-adjusted hematological parameters were positively associated with BMI in linear regression (P<0.05), but not after adjustment for HOMA or waist circumference (WC) and potential metabolic confounders. No associations were found between hematological parameters and BMI in lean, overweight or obese subgroups. Associations between hematological parameters and HOMA were conversely independent of BMI in all subjects and in lean and overweight subgroups (P<0.01), but not in obese subjects alone.

**Conclusions:**

In a North-East Italy general population cohort, obesity per se is not independently associated with altered RBC, Hb and Ht, and the association between BMI and hematological parameters is mediated by their associations with abdominal fat and insulin resistance markers. High hematological parameters could contribute to identify insulin resistance in non-obese individual, but they do not appear to be reliable insulin resistance biomarkers in obese subjects.

## Introduction

Obesity is a major risk factor for insulin resistance [1], [2], but factors linking obesity and increased insulin resistance are not completely understood [1]–[3]. High red blood cells and hematocrit have been hypothesized to contribute to insulin resistance through rheological alterations and impaired tissue blood flow [4], [5], and red blood cell count, hematocrit and hemoglobin have been reported to be positively associated with insulin resistance [6]–[10]. Available studies in general population cohorts are however limited, and potential interactions between obesity, hematological parameters and insulin resistance remain undefined. In particular, evidence is lacking on the potential impact of obesity per se on hematological parameters and on potential involvement of hematological parameters in obesity-associated insulin resistance. We therefore investigated the associations between body mass index (BMI), hematological parameters and insulin resistance, calculated using the validated homeostasis model assessment (HOMA) index [11], in a cohort from the MoMa study, an epidemiological investigation aimed at assessing the prevalence of obesity in the North-East Italy Friuli-Venezia Giulia Region. In the whole cohort and in lean, overweight or obese individuals, we specifically sought to determine whether or not 1) BMI and obesity per se are independently associated with higher RBC, hemoglobin and hematocrit and 2) hematological parameters are potential independent predictors of insulin resistance in obese individuals.

## Methods and Procedures

### Experimental protocol and study population

The MoMa study population was recruited in the municipalities of Montereale Valcellina and Maniago, Pordenone, in the Friuli-Venezia Giulia Region in North-East Italy. The study was conducted by the University of Trieste, in collaboration with local General Practitioners and nurses in the GP outpatient clinic, in the setting of the Friuli-Venezia Giulia Region Prevention Project for Metabolic and Cardiovascular Disease. The study protocol was approved by the competent Pordenone Hospital Ethics Committee, and each subject gave written informed consent to participate before recruitment. A random sample of 2500 18–69 year-old residents in the two municipalities was invited to participate, and 1836 accepted the invitation (M/F = 865/971) and were studied between 2008–2010.

The study protocol included collection of clinical history, medical examination and blood sampling after a 10–12 hour overnight fast. Weight and height were measured and recorded to the nearest 0.1 kg and 0.5 cm, respectively. BMI was calculated as weight in kilograms divided by height in meters squared. Waist circumference was measured on bare skin during midrespiration at the natural indentation between the 10th rib and iliac crest to the nearest 0.5 cm. Blood pressure was measured on the right and left arms using a standard mercury sphygmomanometer. All variables were measured in duplicate, and the average of two measures was used for patient classification. Mean arterial pressure (MAP) was calculated as DBP + 1/3 Diff-BP, where DBP and Diff-BP are diastolic blood pressure and differential blood pressure, respectively. Collected plasma aliquots were used to measure routine biochemical profiles at the Pordenone Hospital Laboratory, and for storage at −80°C until further analyses in the Internal Medicine laboratories at the University of Trieste. For multiple regression models, definitions of metabolic alterations were based on metabolic syndrome thresholds in ATP III classification. Hypertension was therefore defined as systolic or diastolic blood pressure ≥135 and ≥85 mmHg, respectively, or use of antihypertensive medications; hypertriglyceridemia was defined as plasma triglycerides ≥150 mg/dl or use of lipid-lowering drugs; low HDL-cholesterol was defined as plasma HDL-cholesterol <50 or 40 mg/dl in female and male sex respectively; hyperglycemia was defined as plasma glucose ≥110 mg/dl or use of antidiabetic medications; high waist circumference was defined as waist circumference >88 or 102 cm in female and male sex respectively. Hypercholesterolemia was further defined as plasma total cholesterol >200 mg/dl or use of cholesterol-lowering drugs, and diabetes mellitus was diagnosed based on fasting plasma glucose higher than 126 mg/dl or antidiabetic medications.

### Plasma metabolic profile and homa index

RBC count, Hb and Ht, plasma glucose, insulin, triglycerides, total and high-density lipoprotein (HDL) cholesterol were measured using standard methods. Homeostasis model assessment (HOMA) validated index of insulin resistance [11] was calculated with the following formula: HOMA  =  (FPG × FPI)/22.5, where FPG and FPI are fasting plasma glucose (mmol) and fasting plasma insulin (µU/ml), respectively.

### Statistical analysis

Data distributions for continuous variables was assessed by Shapiro-Wilk test and appropriate transformations used for non normal data. Associations between hematological parameters and other variables were evaluated by Pearson correlation. Parameters showing univariate association (p<0.05) were included in stepwise multiple linear regression models in order to assess their impact in the relationship between BMI, HOMA and hematological parameters in the whole population and in BMI subgroups. Multiple linear regression analysis was validated by assessing the normality of residuals. In subgroup analysis, since neither hematological measurements nor their transformations showed normal distribution, differences were evaluated by Kruskal–Wallis tests followed by post-hoc Mann-Whitney comparisons. P values were corrected for multiple comparisons according to Benjamini and Hochberg. P values <0.05 were considered statistically significant. Analysis was performed by SPSS v.17 software (SPSS, Inc., Chicago, IL). Continuous variables are presented as mean±standard deviation.

## Results

### Anthropometric parameters, clinical and biochemical profile and linear regression analysis (Tables 1,2)

Anthropometric and metabolic profile, prevalence of high blood pressure and diabetes and related medications are reported in Table 1. In all subjects (n = 1836), RBC, Hb or Ht were associated with gender (r = 0.53, 0.65, 0.61; all p<0.01), age (r = −0.05, 0.05, 0.07; all p<0.05) and smoking habits (r = −0.05, 0.06, 0.09; all p<0.05). Sex-, age- and smoking habits-adjusted RBC, Hb and Ht were therefore used in all subsequent analyses. Sex-, age- and smoking habits-adjusted RBC, Hb and Ht were positively associated with BMI (P<0.05). Similar associations were observed between hematological parameters and HOMA (P<0.001) and WC (P<0.01). In addition, all hematological parameters were associated with plasma total cholesterol and blood pressure (P<0.01), while less strong or incomplete associations were observed with plasma glucose, triglycerides and HDL-cholesterol (Table 2).

**Table 1:** Clinical and Biochemical profile.

**Gender (M/F)**	865/971
**Age (years)**	49±13 (18–73)
**BMI (kg/m^2^)**	26.9±5.2 (18.6–52.1)
**WC (cm)**	93.2±13.7 (57.0–152.0)
**Prevalence (%) Metabolic Syndrome**	26,7
**Diabetes (Pharm. Tx)**	8.8 (4.5)
**Hypertension (Pharm Tx)**	48.5 (24.3)
**Smoking**	21,4
**SBP (mmHg)**	135±18 (80–215)
**DBP (mmHg)**	81±10 (47–121)
**RBC (RBC/mm^3^)**	4.79±0.41 (3.71–7.02)
**Hb (g/dl)**	14.3±1.2 (9.1–18.3)
**Ht (%)**	42.4±3.4 (31.0–53.6)
**Glucose (mmol/l)**	5.55±1.33 (3.83–21.92)
**Insulin (µU/ml)**	10.2±7.5 (2.0–96.1)
**HOMA**	2.63±2.66 (0.4–63.7)
**Tg (mmol/l)**	1.35±0.91 (0.34–13.76)
**T-Chol (mmol/l)**	5.61±1.10 (2.43–10.06)
**HDL-Chol (mmol/l)**	1.47±0.36 (0.57–3.05)

Gender, age, body mass index (BMI), waist circumference (WC), disease (percent of pharmacological treatment: Tx) and smoking prevalence, systolic (SBP) and diastolic (DBP) blood pressure, red blood cells (RBC), hemoglobin (Hb), hematocrit (Ht), plasma glucose, insulin, homeostasis model assessment (HOMA), plasma triglycerides (Tg), total (T-Chol) and high-density-lipoprotein (HDL-Chol) cholesterol in the whole study population. Results are reported as Mean ± SD (Range).

**Table 2:** Linear regression analyses.

	adj RBC	adj Ht	adj Hb
BMI	0.091***	0.046*	0.055*
WC	0.090***	0.064**	0.089***
SBP	0.061**	0.065**	0.071**
DBP	0.105***	0.105***	0.128***
MAP	0.093**	0.095**	0.111**
Glucose	0.056*	0.026	0.066**
Insulin	0.111***	0.100***	0.118***
HOMA	0.089***	0.074***	0.102***
Tg	0.042	0.053*	0.102***
T-Chol	0.071**	0.120***	0.145***
HDL-Chol	−0.082***	0.037	0.003

Linear regression analyses between age-, sex- and smoking-adjusted (adj) red blood cells (RBC), hemoglobin (Hb), hematocrit (Ht) and body mass index (BMI), waist circumference (WC), systolic (SBP), diastolic (DBP) and mean (MAP) arterial blood pressure, plasma glucose, insulin, homeostasis model assessment (HOMA), plasma triglycerides (Tg), total (T-Chol) and high-density-lipoprotein (HDL-Chol) cholesterol in the whole study population. *:p<0.05,**:p<0.01, ***:p<0.001.

### Multiple regression analyses between BMI and hematological parameters and BMI subgroup analyses - (Figure 1, Tables 3,4)

To determine whether the associations between BMI and RBC, Hb and Ht were independent of related metabolic variables, multiple regression analyses were performed including HOMA, WC, MAP, plasma triglyceride, total and HDL-cholesterol and glucose. The associations between BMI and hematological parameters were no longer statistically significant or became substantially weaker after adjusting for HOMA (Table 3), and they were also not statistically significant after adjusting for WC or in a model including combined metabolic parameters (Table 3).

**Table 3:** Multiple regression analyses.

		RBC			Ht			Hb		
		β (95%C.I.)	F	R^2^	β (95%C.I.)	F	R^2^	β (95%C.I.)	F	R^2^
**BMI**	Model 1	1.35 (0.67–2.02)***	15.3	0.01	0.08 (0–0.17)*	4.0	0.00	0.30 (0.05–0.55)*	5.7	0.00
	Model 2a	0.70 (0.11–1.28)*	306.4	0.25	0.01 (−0.05–0.09)	302.9	0.25	0.02 (−0.19–0.24)	302.9	0.25
	Model 3	0.21 (−0.14–0.56)	2582.5	0.74	−0.02 (−0.06–0.03)	2580.9	0.74	−0.11 (−0.24–0.01)	2585.7	0.74
	Model4	0.56 (−0.05–1.17)	82.0	0.21	0.03 (−0.05–0.11)	81.5	0.21	0.01 (−0.21–0.24)	81.3	0.21
	Model5	0.13 (−0.22–0.48)	762.1	0.75	−0.02 (−0.06–0.03)	762.1	0.75	0.02 (−0.2–0.25)	763.9	0.74
	Model6	0.41 (−0.11–0.93)	193.4	0.43	0.01 (−0.06–0.08)	192.9	0.43	−0.03 (−0.22–0.17)	192.8	0.43
**HOMA**	Model 1	0.68 (0.33–1.03)***	14.5	0.01	0.07 (0.02–0.12)**	10.2	0.01	0.28 (0.15–0.41)***	19.2	0.01
	Model 2b	0.34 (0.03–0.64)*	305.9	0.25	0.05 (0.01–0.09)*	307.2	0.25	0.21 (0.10–0.32)***	311.8	0.25
	Model 3	0.37 (0.05–0.68)*	250.7	0.22	0.05 (0.00–0.09)*	250.4	0.22	0.17 (0.06–0.29)**	252.9	0.22
	Model4	0.40 (0.10–0.70)**	124.1	0.29	0.06 (0.02–0.10)**	124.8	0.29	0.17 (0.06–0.28)**	124.7	0.29
	Model5	0.31 (0.03–0.60)*	154.3	0.37	0.05 (0.02–0.09)**	155.0	0.37	0.14 (0.04–0.25)**	154.9	0.37
	Model6	0.41 (0.09–0.72)*	69.6	0.21	0.06 (0.02–0.10)**	69.9	0.21	0.20 (0.08–0.31)**	70.4	0.21

Multiple regression analyses between red blood cells (RBC), hemoglobin (Hb), hematocrit (Ht) (independent variables) and body mass index (BMI) or homeostasis model assessment (HOMA) (dependent variables) in the whole study population (n = 1836). Similar results were observed when waist circumference (WC) was included instead of BMI (not shown). *: p<0.05; **: p<0.01; ***: p<0.001. P value for F test was <0.001 in all models except for Model 1 with BMI dependent variable (P<0.05).

Data adjustments:

Model 1: age, sex, smoking habits.

Model 2a: Model 1 + HOMA.

Model 2b: Model 1 + BMI.

Model 3: Model 1 + Waist Circumference.

Model 4: Model 1 + Triglycerides, total cholesterol, HDL cholesterol, Mean Arterial Pressure, Glucose.

Model 5: Model 4 + WC.

Model 6: Model 1 + Hypertriglyceridemia, Hypercholesterolemia, Low HDL, Hypertension, Hyperglycemia, High WC.

**Figure 1:**
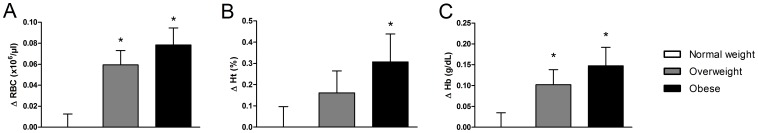
Changes in red blood cells (RBC) (A), hematocrit (Ht) (B) and hemoglobin (Hb) (C) in overweight (OW) and obese relative to Lean body mass index subgroups. *: p<0.05 vs. Lean.

In BMI subgroup analyses, lowest RBC, Hb and Ht were observed in lean subjects (P<0.05), but no stepwise increments were observed from overweight to obese BMI groups (P = NS overweight vs obese, Figure 1). Similar patterns were observed for WC tertiles (P = NS Middle vs High WC, not shown). No statistically significant associations were finally observed in linear regression analysis between BMI and sex-, age- and smoking-adjusted hematological parameters in each BMI group considered separately (Table 4).

**Table 4:** Multiple regression analyses.

		RBC			Ht			Hb		
BMI		β (95%C.I.)	F	R^2^	β (95%C.I.)	F	R^2^	β (95%C.I.)	F	R^2^
**Lean**	Model 1	0.29 (−0.08–0.67)	2.4	0.00	−0.02 (−0.06–0.03)	0.4	0.00	−0.02 (−0.15–0.12)	0.1	0.00
	Model 2	0.05 (−0.28–0.37)	120.1	0.25	−0.04 (−0.08–0.00)	122.3	0.25	−0.13 (−0.24–0.01)	123.0	0.25
	Model 3	0.17 (−0.20–0.54)	16.9	0.05	−0.03 (−0.08–0.02)	17.4	0.05	−0.07 (−0.21–0.06)	17.1	0.05
**OW**	Model 1	0.07 (−0.23–0.36)	0.2	0.00	0.00 (−0.04–0.04)	0.0	0.00	0.07 (−0.04–0.18)	1.7	0.00
	Model 2	0.09 (−0.17–0.35)	94.8	0.22	0.01 (−0.03–0.04)	94.6	0.22	0.04 (−0.06–0.14)	95.0	0.22
	Model 3	−0.01 (−0.30–0.28)	18.1	0.05	−0.01 (−0.05–0.03)	18.2	0.05	0.02 (−0.09–0.13)	18.2	0.05
**Obese**	Model 1	0.30 (−0.89–1.48)	0.2	0.00	0.08 (−0.07–0.23)	1.1	0.00	−0.05 (−0.48–0.38)	0.1	0.00
	Model 2	0.31 (−0.48–1.09)	273.3	0.57	−0.02 (−0.12–0.08)	272.7	0.57	−0.14 (−0.42–0.15)	273.6	0.57
	Model 3	0.07 (−1.05–1.19)	28.4	0.12	0.06 (−0.08–0.20)	28.7	0.12	−0.27 (−0.56–0.25)	28.7	0.12

Multiple regression analyses between red blood cells (RBC), hemoglobin (Hb), hematocrit (Ht) and body mass index (BMI) in normal weight (Lean, n = 725), overweight (OW, n = 688), obese (Obese, n = 423) subjects from the study population (n = 1836). P value for F test was <0.001 in all models except for Model 1.

Data adjustments:

Model 1: age, sex, smoking habits.

Model 2: Model 1 + Waist Circumference.

Model 3: Model 1 + Homeostasis Model Assessment.

### Multiple regression analyses between hematological parameters and HOMA and BMI subgroup analyses (Tables 3,5)

To determine whether the associations between hematological parameters and HOMA index were independent of BMI and waist circumference, multiple regression analyses were performed. The associations between RBC, Hb, Ht and HOMA remained statistically significant after adjustment for BMI or WC as well as combined metabolic variables (Table 3).

In BMI subgroup analyses, HOMA values expectedly increased from lean to overweight to obese individuals (Lean: 1.63±0.03, Overweight: 2.50±0.06, Obese: 4.57±0.22, P<0.01). The association between hematological parameters and HOMA remained statistically significant and independent of BMI or WC only in lean and overweight subjects (P<0.05) while not in the obese patient group (P = NS) (Table 5).

**Table 5:** Multiple regression analyses.

		RBC			Ht			Hb		
HOMA		β (95%C.I.)	F	R^2^	β (95%C.I.)	F	R^2^	β (95%C.I.)	F	R^2^
**Lean**	Model 1	0.31 (0.13–0.49)***	11.6	0.02	0.04 (0.02–0.06)***	10.4	0.01	0.13 (0.06–0.19)***	15.1	0.02
	Model 2	0.28 (0.11–0.46)**	21.6	0.06	0.04 (0.02–0.06)***	22.6	0.06	0.13 (0.07–0.19)***	24.9	0.07
	Model 3	0.26 (0.09–0.44)**	21.6	0.06	0.03 (0.01–0.06)**	21.6	0.06	0.11 (0.04–0.17)***	22.9	0.06
**OW**	Model 1	0.38 (0.03–0.73)*	4.6	0.01	0.07 (0.02–0.11)**	8.0	0.01	0.27 (0.14–0.40)***	16.0	0.02
	Model 2	0.37 (0.03–0.71)*	20.4	0.06	0.07 (0.02–0.11)**	22.5	0.06	0.25 (0.12–0.38)***	25.7	0.07
	Model 3	0.40 (0.05–0.74)*	14.0	0.04	0.07 (0.02–0.11)**	15.9	0.04	0.25 (0.13–0.38)***	19.1	0.05
**Obese**	Model 1	0.68 (−0.60–1.95)	1.1	0.00	0.07 (−0.09–0.23)	0.7	0.00	0.31 (−0.15–0.76)	1.7	0.00
	Model 2	0.57 (−0.63–1.77)	28.9	0.12	0.04 (−0.11–0.19)	28.5	0.12	0.33 (−0.11–0.76)	29.6	0.12
	Model 3	0.68 (−0.53–1.88)	26.6	0.11	0.02 (−0.13–0.17)	26.0	0.11	0.27 (−0.17–0.70)	26.7	0.11

Multiple regression analyses between red blood cells (RBC), hemoglobin (Hb), hematocrit (Ht) and homeostasis model assessment (HOMA) in normal weight (Lean, n = 725), overweight (OW, n = 688), obese (Obese, n = 423) subjects from the study population (n = 1836). *: p<0.05; **: p<0.01; ***: p<0.001. P value for F test was <0.01 in all models except for Model 1 in the Obese group.

Data adjustments:

Model 1: age, sex, smoking habits.

Model 2: Model 1 + Body Mass Index.

Model 3: Model 1 + Waist Circumference.

### Multiple regression analyses between hematological parameters and blood pressure, plasma glucose, triglycerides and cholesterol (Table 6)

**Table 6:** Multiple regression analyses.

		RBC			Ht			Hb		
		β (95%C.I.)	F	R^2^	β (95%C.I.)	F	R^2^	β (95%C.I.)	F	R^2^
**Model 1**	MAP	0.002 (0.001–0.003)**	8.8	0.02	0.016 (0.006–0.027)**	7.4	0.02	0.006 (0.002–0.009)**	11.7	0.03
	Glucose	0.000 (0.000–0.001)			0.001 (−0.005–0.006)			0.001 (0.000–0.003)		
	Tg	0.000 (0.000–0.000)			0.000 (−0.002–0.002)			0.000 (0.000–0.001)		
	T-Chol	0.001 (0.000–0.001)***			0.006 (0.003–0.010)***			0.003 (0.001–0.004)***		
	HDL-Chol	−0.003 (−0.004–0.002)***			0.002 (−0.008–0.012)			−0.001 (−0.005–0.002)		
**Model 2**	MAP	0.002 (0.000–0.003)*	7.9	0.03	0.015 (0.004–0.026)**	6.2	0.02	0.006 (0.002–0.009)**	9.7	0.03
	Glucose	0.000 (0.000–0.001)			0.000 (−0.005–0.006)			0.001 (0.000–0.003)		
	Tg	0.000 (0.000–0.000)			0.000 (−0.002–0.002)			0.000 (0.000–0.001)		
	T-Chol	0.001 (0.000–0.001)***			0.006 (0.003–0.010)***			0.003 (0.001–0.004)***		
	HDL-Chol	−0.003 (−0.004–0.001)***			0.003 (−0.007–0.013)			−0.001 (−0.005–0.003)		
**Model 3**	MAP	0.002 (0.000–0.003)*	8.4	0.03	0.015 (0.004–0.026)**	7.8	0.02	0.005 (0.001–0.009)**	11.4	0.04
	Glucose	0.000 (−0.001–0.001)			−0.003 (−0.009–0.002)			0.000 (−0.002–0.002)		
	Tg	0.000 (−0.001–0.000)			0.000 (−0.002–0.001)			0.000 (0.000–0.001)		
	T-Chol	0.001 (0.000–0.001)***			0.007 (0.003–0.010)***			0.003 (0.002–0.004)***		
	HDL-Chol	−0.003 (−0.004–0.002)***			0.004 (−0.006–0.014)			−0.001 (−0.004–0.003)		
**Model 4**	MAP	0.002 (0.000–0.003)*	7.3	0.03	0.014 (0.003–0.026)*	6.7	0.02	0.006 (0.002–0.01)**	10	0.04
	Glucose	0.000 (−0.001–0.001)			−0.003 (−0.009–0.002)			0.000 (−0.002–0.002)		
	Tg	0.000 (−0.001–0.000)			0.000 (−0.002–0.001)			0.000 (0.000–0.001)		
	T-Chol	0.001 (0.000–0.001)***			0.007 (0.003–0.010)***			0.003 (0.002–0.004)***		
	HDL-Chol	−0.003 (−0.004–0.001)***			0.004 (−0.006–0.014)			−0.001 (−0.005–0.003)		

Multiple regression analyses between mean arterial pressure (MAP), plasma glucose, triglycerides (Tg), total (T-Chol) or HDL-cholesterol (HDL-Chol) and red blood cells (RBC), hematocrit (Ht) and hemoglobin (Hb) in the whole study population (n = 1836). *: p<0.05; **: p<0.01; ***: p<0.001. P value for F test was <0.001 in all models. Similar results were observed when WC was included instead of BMI (not shown).

Data adjustments:

Model 1: age, gender, smoking habits.

Model 2: Model 1 + Body Mass Index.

Model 3: Model 1 + Homeostasis Model Assessment (HOMA).

Model 4: Model 2 + HOMA.

To further investigate the potential interactions between blood pressure, plasma glucose, plasma triglycerides, total and HDL cholesterol, as well as their potential independent association with hematological parameters, multiple regressions analyses were performed including BMI or waist circumference and HOMA. The data showed that linear association between hematological parameters and blood pressure were independent of BMI, HOMA or combined HOMA and BMI, and similar results were obtained when waist circumference was used instead of BMI values. Associations with plasma glucose were on the other hand not significant in any model. After adjustments, associations between hematological parameters and lipid profile were statistically significant for total cholesterol, while associations with HDL-cholesterol were only preserved for RBC, while not for Ht or Hb. No statistically significant associations were finally observed between plasma triglycerides and any hematological variable.

## Discussion

The current study investigated the interactions between BMI, hematological parameters and insulin resistance. In a North-East Italy population study cohort, we demonstrated that: 1) a positive association between BMI and red blood cells, hemoglobin and hematocrit is dependent on abdominal fat distribution and insulin resistance markers, and obesity per se is not associated with altered hematological parameters; 2) hematological parameters are associated with insulin resistance independently of anthropometric variables; however, when considering separate BMI subgroups, statistically significant associations between hematological parameters and insulin resistance are observed only in lean and overweight and not in obese BMI subgroups.

The study demonstrates that BMI or obesity per se are not independently associated with higher hematological parameters. This conclusion is directly supported by lack of stepwise increments in red blood cells, hematocrit or hemoglobin in BMI subgroup analyses, and by lack of associations between hematological variables and BMI in different BMI subgroups. In the whole study cohort, red blood cells, hemoglobin and hematocrit are also not associated with BMI after adjusting for insulin resistance and abdominal fat markers, and the general association between BMI and hematological parameters appears therefore to be at least in part dependent on the higher incidence of excess abdominal fat and insulin resistance in both overweight and obese individuals.

Previous reports described positive associations between insulin resistance and red blood cells count or elevated hematocrit [6]–[10], but few studies investigated general population cohorts, and interactions between BMI, hematological parameters and insulin resistance had not been defined [8], [9]. A direct association between insulin resistance and hematological parameters in the current general population was importantly independent of BMI and abdominal fat markers, and excess viscosity in subjects with higher red blood cells and hematocrit could indeed primarily impair insulin action through reduced blood flow in insulin target tissues [4], [5]. Insulin resistance and secondary hyperinsulinemia could however also directly lead to higher red blood cells, since insulin and erythropoietin have synergistic effects in stimulating erythroid colony proliferation [12]. Based on available information, it is therefore possible that a vicious cycle mutually enhances insulin resistance and hematological parameters. The current study also investigated whether hematological parameters are associated with, and therefore represent potential markers of, insulin resistance in different BMI subgroups. BMI subgroup analyses revealed that associations between HOMA and red blood cells, hemoglobin and hematocrit remain statistically significant in lean and overweight but not in obese subjects. Since the obese individuals expectedly had markedly higher insulin resistance, it is likely that lack of associations in this group is due at least in part to a relevant negative impact on insulin action by non-hematological, obesity-associated alterations, that could include altered adipokine profiles, excess substrate availability, oxidative stress and inflammation [1]–[3]. The above observations have potentially relevant clinical implications since they suggest that: 1) although many important factors contribute to modulate insulin resistance, the current data suggest that, along with other parameters, higher hematological parameters may contribute to identify non-obese individuals at early stages of insulin resistance; 2) red blood cells, hemoglobin and hematocrit appear to have a less relevant metabolic impact and are unlikely to represent reliable biomarkers of insulin resistance in obese individuals.

The study provided the opportunity to directly investigate the associations between hematological parameters and obesity-associated metabolic complications. Linear associations were observed between hematological parameters and blood pressure, plasma total and HDL-cholesterol, triglycerides and glucose, with closest associations for blood pressure and lipid profile. Blood pressure remained significantly associated with hematological parameters in multiple regression models, thereby indicating that the previously reported contribution of altered rheological characteristics to enhance blood pressure [13] is at least in part independent of obesity. After adjustments, no associations were instead observed for plasma glucose, while total cholesterol retained the strongest independent association with red blood cells, hemoglobin and hematocrit among lipid variables. This pattern of associations is in general agreement with previous studies [13] and a close association between non-HDL cholesterol and hematological parameters has been recently described in a US population study [14]. The current data therefore contribute to identify an emerging cluster of cardiovascular risk factors including hematological parameters, insulin resistance, blood pressure and total cholesterol, that appears to be at least in part independent of obesity. Further studies will be needed to investigate the mechanisms underlying these relationships.

In conclusion, obesity per se is not associated with high RBC, hemoglobin and hematocrit in a North-East Italy population, and abdominal fat accumulation and insulin resistance appear to mediate a general association between BMI and hematological parameters. High RBC, hemoglobin and hematocrit could contribute to identify insulin resistance in non-obese individuals, but they are unlikely to substantially contribute to insulin resistance and they do not appear to be reliable biomarkers of insulin resistance in obese subjects.
